# Access to Vaccination Information and Confidence/Hesitancy towards Childhood Vaccination: A Cross-Sectional Survey in China

**DOI:** 10.3390/vaccines9030201

**Published:** 2021-02-28

**Authors:** Fanxing Du, Tracey Chantler, Mark R. Francis, Fiona Yueqian Sun, Xuan Zhang, Kaiyi Han, Lance Rodewald, Hongjie Yu, Shiyi Tu, Heidi Larson, Zhiyuan Hou

**Affiliations:** 1 NHC Key Laboratory of Health Technology Assessment, School of Public Health, Fudan University, Shanghai 200032, China; fdu18@fudan.edu.cn (F.D.); Kaiyi.Han@lshtm.ac.uk (K.H.); yhj@fudan.edu.cn (H.Y.); sytu@fudan.edu.cn (S.T.); 2 Department of Global Health and Development, Faculty of Public Health and Policy, London School of Hygiene & Tropical Medicine, London WC1H 9SH, UK; Tracey.Chantler@lshtm.ac.uk; 3 Department of Infectious Disease Epidemiology, London School of Hygiene & Tropical Medicine, London WC1E 7HT, UK; Mark.Francis@lshtm.ac.uk (M.R.F.); Fiona.Sun@lshtm.ac.uk (F.Y.S.); Heidi.Larson@lshtm.ac.uk (H.L.); 4 Chinese Center for Disease Control and Prevention, Beijing 102200, China; zhangxuan@chinacdc.cn (X.Z.); rodewaldl@chinacdc.cn (L.R.); 5 Key Laboratory of Public Health Safety (Ministry of Education), Fudan University, Shanghai 200032, China

**Keywords:** vaccine, confidence, hesitancy, vaccination information, China

## Abstract

Access to vaccination information could influence public attitudes towards vaccination. This study investigated the number and types of vaccination-related information sources, and estimated their associations with vaccine confidence and hesitancy in China. In January 2019, we conducted a cross-sectional survey in China, and 2122 caregivers with children <6 years completed self-administered questionnaires. Logistic regressions were used to assess associations between caregivers’ primary information sources and vaccine confidence/hesitancy. A majority (72%) of caregivers had multiple sources of vaccination-related information. The proportions of caregivers reporting professional sources, media, and peers as primary information sources were 81%, 63%, and 26%. Internal migrants were less likely to get information from professional sources; more educated and wealthier caregivers reported more information sources and were more likely to get information from media and peers. Caregivers who reported professional information sources had significantly higher odds of being confident about the safety of vaccines and lower odds of being hesitant toward vaccination than those who did not. Caregivers who reported the media as a primary information source had significantly higher odds of being hesitant toward vaccination than those who did not. To address vaccine hesitancy, it is essential to promote universal access to professional vaccination-related information sources, and to use the media to disseminate evidence-based information and clarify misinformation. Health communication should target internal migrants, and more educated and wealthier caregivers.

## 1. Introduction

Vaccination is recognized as an effective public health intervention [1,2,3], however this success is being challenged by waning vaccine confidence and growing vaccine hesitancy [4,5]. Access to appropriate information is essential to guide vaccination decisions, especially given the increased circulation of misinformation and negative information about vaccines and the proliferation of anti-vaccination movements [6]. In Japan, misinformation about adverse reactions following the human papillomavirus vaccination spread globally across media platforms in 2013, significantly undermining the uptake of this vaccine [7]. In Italy, misinformation about the measles–mumps–rubella vaccine and autism on the internet was associated with reduced vaccination coverage during 2010–2015 [8]. In China, having heard negative information about vaccines was found to be associated with delaying or refusing childhood vaccination [9]. In Malaysia, anti-vaccination movements can contribute to vaccine hesitancy [10]. Vaccination-related information environments vary across countries and time; hence, it is necessary to understand public exposure to and engagement with vaccination-related information in their local contexts [11,12,13].

The accuracy and reliability of vaccination information sources can be variable. Health professionals are regarded as the most reliable information sources [14,15], while information from media and interpersonal sources may be ambiguous. Technological advances have resulted in people accessing vaccination information from the internet and social media platforms [16]. These platforms can help disseminate evidence-based information [17,18], but have also been implicated in the spread of vaccination-related misinformation [19]. A systematic review of studies that examined how traditional media portrayed immunization reported that 10 in 12 studies observed that media reports conveyed more negative vaccination messages than positive ones, and 6 of 8 studies highlighted that media reports often included inaccurate vaccine information [20]. Exposure to media information was reported to promote vaccination in Asian countries [21], but increase the possibility of vaccine hesitancy in Italy, Botswana, the Dominican Republic, and Greece [11,22]. Interpersonal information sources, such as family members or friends, may also influence health beliefs towards vaccination differently. A negative relationship between interpersonal information sources and HPV vaccine acceptance was reported in Italy [23], whereas interpersonal information sources were effective to improve health beliefs towards vaccination in Korea and the United States [24,25]. These inconsistent findings may in part be due to the content of information shared and the local contexts in each country. Thus, the influence of information sources on vaccination needs to be studied in local contexts, which can help determine the locally relevant information sources and guide tailored health communication interventions to the public.

Vaccine incidents involving coincidental, non-vaccine-related death or disability, such as coincidental childhood death or disability following vaccination in Shanxi province in 2010 [26], coincidental infant death following hepatitis B vaccination in Hunan province in 2013 [27], illegal sale of vaccines in Shandong province in 2016 [28], and the production of substandard vaccines by Changchun Changsheng Biotechnology Company in 2018 [29], have undermined the public’s confidence in and acceptance of vaccines in China [9,29,30]. The delivery of transparent and accurate information is critical to rebuild vaccine confidence and maintain high vaccination coverage following such vaccine incidents. This study aimed to investigate the number and types of primary sources of vaccination-related information the public accesses, and estimate associations between the reported primary information sources and vaccine confidence and hesitancy in China.

## 2. Materials and Methods

### 2.1. Data Collection

**Figure A1:**
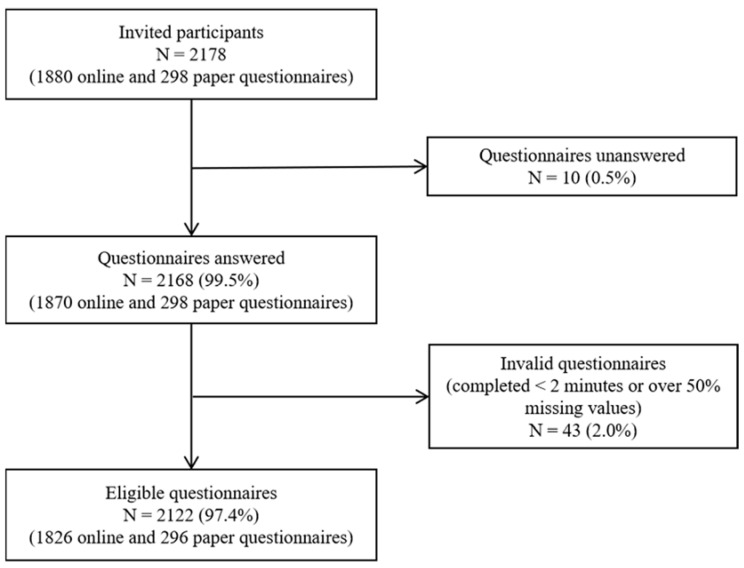
The process of participant recruitment.

In January 2019, we conducted a cross-sectional survey in Shaanxi province, Anhui province, and Shenzhen megacity in Guangdong province, located in Western, Central, and Eastern China, respectively. A two-stage, cluster-sampling process was used to enroll caregivers of children < 6 years old: we selected totally three urban districts and two rural counties in the three provinces, and 3 or 4 communities were selected according to their socioeconomic status in each district/county. In each sampled community, all caregivers were recruited from one vaccination clinic and one kindergarten respectively. Caregivers of all children visiting the sampled vaccination clinics on a given day during the survey period and from a class in the sampled kindergartens were invited to participate in our survey. Additional details on the sampling and recruitment methodology is reported elsewhere [31,32]. Caregivers were invited to complete questionnaires—by themselves, on their mobile phones, or in writing on the spot with assistance after informed consent was obtained. The questionnaires were self-administered and took approximately 10 min to complete. A total of 2178 caregivers provided oral informed consent and were invited to participate in the questionnaire survey, and after removing unanswered, incomplete, and invalid questionnaires, the final sample comprised 2122 participants. The process of participant recruitment is shown in Figure A1 in Appendix A. The Fudan University School of Public Health and the London School of Hygiene & Tropical Medicine Ethics committees approved the study protocol (FDU IRB#2018-10-0703, LSHTM Ethics Ref 160160).

### 2.2. Measures

Caregivers were asked to select their primary sources of information about childhood vaccination from the following items: (1) health professionals; (2) health education activities, and materials (i.e., brochures and leaflets) about vaccination; (3) professional books about vaccination; (4) internet or social media; (5) traditional media; (6) friends or colleagues; and (7) family members or relatives. They were required to select at least one source and at most three sources. To measure the number of caregivers’ primary information sources, we counted how many items they selected from the seven items above. In order to measure the types of caregivers’ primary information sources, we built three dichotomous variables: “professional sources” (have/do not have), “media sources” (have/do not have), and “interpersonal sources” (have/do not have). If the choices of a respondent include at least one of the following three items: health professionals, health education activities and materials, and professional books, it means he/she has “professional sources”, otherwise he/she does not have. If the choices of a respondent include at least one of the two sources: internet or social media, and traditional media, it means he/she has “media sources”, otherwise he/she does not have. If the choices of a respondent include one or two of the sources: friends or colleagues, and family members or relatives, it means he/she has “interpersonal sources”, otherwise he/she does not have.

The assessment of caregivers’ vaccine confidence covered three domains—confidence in safety of childhood vaccines, confidence in effectiveness of childhood vaccines, and confidence in health professionals. These domains were measured by the extent to which caregivers agreed with the following statements on a five-point Likert scale: “overall vaccines are safe”, “overall vaccines are effective”, and “I trust the advice and information on vaccination from health professionals.” [4] Responses were grouped into two categories: agree (including “strongly agree” and “tend to agree”) and disagree (including “strongly disagree”, “tend to disagree” and “neutral or don’t know”). Vaccine hesitancy was measured using questions based on the World Health Organization Strategic Advisory Group of Experts on Immunization definition [33]: “have you ever hesitated, delayed, or refused about getting a vaccination for your child due to reasons other than allergies and sickness?” Caregivers who ever hesitated, delayed, or refused getting a vaccination for their child were categorized “hesitant to vaccination”.

### 2.3. Statistical Analysis

Data from the online questionnaires were automatically uploaded to the Wenjuanxing online platform in real time, and data recorded on paper questionnaires were double entered using EpiData. To compare the number and types of primary information sources by participant characteristics, we performed univariate analyses using Chi-square tests or Fisher’s exact tests (if expected frequency < 5) for categorical measures and ANOVA for continuous measures. In addition, univariate analyses and multivariate analyses were performed to investigate associations between caregiver types of primary information sources (professional sources, media sources, and interpersonal sources), and vaccine confidence/hesitancy using Chi-square tests (or Fisher’s exact tests if expected frequency < 5) and logistic regressions. In multivariate analyses, we controlled for demographic and socioeconomic characteristics of caregivers, including region, living residence (rural or urban), gender, age, religious beliefs, education level, annual household income, and registered residence (local resident or internal migrant). Besides, a respondent may have more than one type of information sources, so we put three variables (professional sources, media sources, and interpersonal sources) together in each model to control the effect of the other two types when we assess the influence of one type. Results of the logistic regressions are presented as adjusted odds ratio (AOR) and 95% confidence intervals (CI). A *p* < 0.05 was considered as statistically significant. All statistical analyses were performed using Stata, version 14.0 (StataCorp LP, College Station, TX, USA).

## 3. Results

### 3.1. Sample Characteristics

Of the 2122 caregivers with valid data, participants from Shaanxi province, Anhui province, and Shenzhen city accounted for 40.4%, 40.3%, and 19.3%, respectively (Table 1). Most caregivers were local permanent residents (81.4%), female (76.6%), and had no religious beliefs (92.2%). Almost two-thirds lived in urban areas (61.0%) and had completed three-year technical college (higher than high school) or university (62.2%). The mean age of caregivers was 34 years with a standard deviation of 8 years.

### 3.2. Sources of Vaccination-Related Information

**Figure 1:**
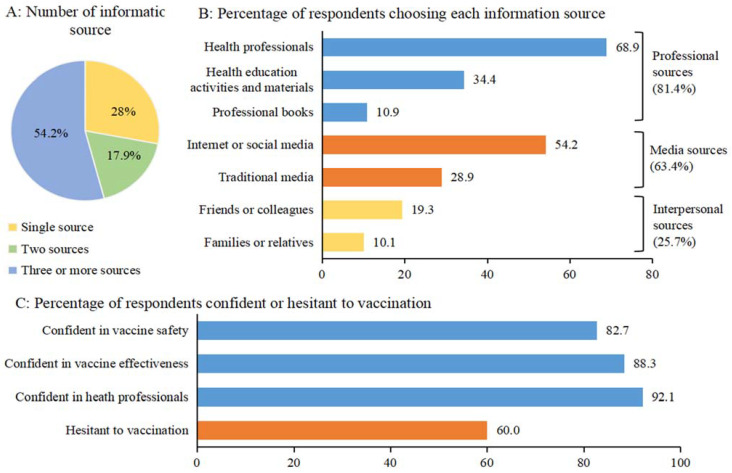
Number and types of vaccination-related information sources, and vaccine confidence/hesitancy.

The proportion of caregivers who reported one, two, or three or more information sources was 28.0% (593/2122), 17.9% (380/2122), and 54.2% (1149/2122), respectively (Figure 1A). The proportions of caregivers reporting each information sources presented in Figure 1B, indicate that health professionals (68.9%, 1462/2122) and the internet or social media (54.2%, 1149/2122) were the top information sources. In total, 81.4% (1728/2122), 63.4% (1346/2122), and 25.7% (546/2122) of caregivers have professional sources, media sources, and interpersonal sources, respectively.

In the univariate analysis (Table 1), caregivers from Shenzhen city reported a significantly higher number of information sources than caregivers from the Anhui and Shaanxi provinces (*p* = 0.047). Caregivers who lived in urban areas, female, younger than 40 years old, educated to a level of three-year technical college or above, and with high income also reported significantly more information sources than their counterparts. In addition, the proportion of caregivers who had professional sources was significantly higher among female caregivers, those with lower middle income (20,000–50,000 RMB), and local residents. The proportion of caregivers who had media sources was significantly higher among those living in urban areas (*p* = 0.003) and younger caregivers (*p* = 0.026). Finally, the proportions of caregivers who had media sources and those who had interpersonal sources were significantly higher among caregivers with higher education and income levels.

### 3.3. Associations between Information Sources and Vaccine Confidence/Hesitancy

A majority of caregivers, 82.7% (1754/2122) and 88.3% (1870/2119) agreed that vaccines were safe and effective, respectively, and 92.1% (1953/2121) reported trusting healthcare professionals (Figure 1C). Over half of the caregivers (60.0%, 1274/2122) reported some hesitation towards vaccinating their children. In the univariate analysis (Table 2), the prevalence of vaccine hesitancy was significantly higher among caregivers who reported three or more primary information sources compared to those reporting only one source (66.6% vs. 45.9%, *p* < 0.001). Furthermore, caregivers who had professional sources had significantly higher confidence in vaccines and health professionals, and lower vaccine hesitancy, whereas caregivers who had media sources and those who had interpersonal sources had significantly lower confidence in vaccines and health professionals, and higher vaccine hesitancy (Table 2).

The multivariate logistic regressions (Table 3) showed that caregivers who reported two and three or more vaccination-related information sources had significantly higher odds of being hesitant to vaccination than those reporting a single information source. Caregivers who reported professional sources as one of their primary vaccination-related information sources had a significantly higher odds of being confident in the safety of vaccines (AOR: 1.68; 95%CI: 1.09–2.59) and lower odds of being vaccine hesitant (AOR: 0.63; 95%CI: 0.45–0.87) than those who did not report using professional sources as primary information sources. Caregivers who had media sources had a significantly higher odds of being hesitant to vaccination (AOR: 1.47; 95%CI: 1.19–1.83) than those who did not have. Reporting peers as one of their primary information sources did not have a significant association with caregivers’ vaccine confidence or hesitancy. In addition, the number and types of caregivers’ primary vaccination-related information sources had no significant association with confidence in the effectiveness of vaccines or in health professionals.

### 3.4. Associations between Demographic/socioeconomic Characteristics and Vaccine Confidence/Hesitancy

According to the multivariate logistic regressions (Table 3), females (AOR: 0.66; 95%CI: 0.48–0.91), and caregivers whose annual household income >200,000 RMB (AOR: 0.65; 95%CI: 0.44-0.94) had lower odds of being confident in vaccine safety compared to their counterparts. Caregivers aged 30–35 years had higher odds of being hesitant to vaccination (AOR: 1.52; 95%CI: 1.22–1.90), while those aged >40 years had lower odds (AOR: 0.54; 95%CI: 0.38–0.76). Caregivers who had Buddhism or other religions (AOR: 1.70; 95%CI: 1.16–2.49) and those educated with three-year technical college (AOR: 1.33; 95%CI: 1.04–1.72) had significantly higher odds of being hesitant compared to their counterparts. Internal migrants had lower odds of being hesitant than local residents (AOR: 0.78; 95%CI: 0.63–0.98).

## 4. Discussion

Our study estimated the number and types of primary vaccination information sources among caregivers and investigated associations between the use of these information sources and vaccine confidence and hesitancy in China. Most caregivers (72%) used multiple sources to access vaccination-related information. Around 81%, 63%, and 26% of caregivers reported professional sources, media, and peers as their primary information sources, respectively. Internal migrants were less likely to report professional sources as primary information sources, and 31% of caregivers did not report health professionals as primary information sources. More educated and wealthier caregivers used a higher number of information sources and were more likely to select the media and their peers as their primary information sources. Prioritizing professional sources to access vaccination information was associated with higher vaccine confidence, whereas caregivers who relied on the media for vaccination information were more likely to report vaccine hesitancy.

This strong association between caregivers using professional information sources and vaccine confidence is consistent with previous research [34,35,36]. Information from professional sources is known to improve the public’s vaccination knowledge and positively influence their perceptions of the need for vaccination [37]. As the prevalence of vaccine-preventable diseases has dramatically reduced due to the success of vaccination programs around the world, the public may underestimate the need for and benefit of vaccination without sufficient access to professional vaccination information sources [37]. Exposure to negative vaccination information and limited vaccination knowledge may further increase the probability of the public’s perceiving vaccination as risky [38]. A global study between 2014 and 2016 indicated that skewed risk-benefit perceptions and a lack of vaccination knowledge were important reasons for vaccine hesitancy among caregivers [5]. In contrast, evidence-based information from professional sources can play a key role in increasing vaccine acceptance by improving caregivers’ understanding of vaccination and vaccine-preventable diseases [15,39]. However, only 69% of caregivers reported health professionals as primary information sources in China, which was 15–20% lower than estimates from developed countries: 84% in France [34], 83% in Australia [40], and 92% in the United States [11]. This suggests a need to increase Chinese caregivers’ access to vaccine information from professional sources. This cannot be achieved without the participation of general practitioners or primary care professionals. Vaccination services are delivered by general practitioners in many countries such as England, however, in China, they are delivered by dedicated vaccinators at vaccination clinics held in community health centers. Community health centers employ 50–100 health professional of which only 3–5 serve as vaccinators, who are generally responsible for tens of thousands of residents. Primary care professionals do not have any vaccination responsibilities and do not play an active role in vaccination related health education or consultations [11]. It is time to address the segmentation of clinical and preventive care, and to encourage primary care professionals to play a greater role in promoting vaccination and helping caregivers access evidence-based and accurate vaccination-related information.

In our study, caregivers who reported the media as one of their primary information sources were more likely to be hesitant towards vaccination, which is consistent with studies from Italy, Botswana, the Dominican Republic, and Greece [11,22]. However, studies in South and Southeast Asia countries have indicated that mothers’ media usage is related to the improved uptake of childhood vaccination [21,41]. The differential influence of media on vaccine hesitancy may depend on the nature of vaccination-related information provided in the media. The media represent a double-edged sword that could disseminate health knowledge to promote beneficial health behaviors but also spread misinformation and negative information [42,43]. Recently, the public has had a higher tendency to obtain information from media, especially through the internet and social media [19], and in our study, internet or social media accounted for the second major source of vaccination-related information (54%) reported by caregivers. Information spreads rapidly on internet and social media, which is especially true for misinformation and negative information [8,44]. According to the social amplification of risk theory [45], misinformation, rumors, and anti-vaccination propaganda have an easier and broader spread than positive and accurate information. In China, recent vaccine incidents [29,30] have led to the spread of negative information, and the public has been exposed to this negative information environment. A previous study has illustrated that the spread of fake news and misinformation on social media is significantly associated with vaccine hesitancy [8], and information sources from the internet and social media were independently associated with a 2.5 times increased likelihood for vaccine hesitancy even when vaccination knowledge was adjusted for [16]. Exposure to negative information may influence perceptions of risk due to vaccination, and these risk perceptions are more likely to be amplified through viral dissemination on social media [45]. In addition, our results showed that caregivers with a higher number of information sources were more likely to have vaccine hesitancy, possibly because information from different sources may be contradictory and cause confusion. Due to the increasing use of media as information sources, health organizations and professionals should take full advantage of various media channels to disseminate evidence-based vaccination-related information and to provide timely clarification of misinformation [43].

In addition, caregivers educated with three-year technical college were more likely to have media sources and interpersonal sources than people with lower education level (Table 1), and they may not have enough knowledge about vaccination comparing to people with bachelor degree or above. That might explain why people in this group had significantly higher odds of being hesitant than others (Table 3). We also found that people with the highest household income levels had more information sources than others, and they were less likely to have professional sources and more likely to have media and interpersonal sources (Table 1). That maybe the reason why caregivers with highest income level were more concerned about vaccine safety than those with lower income levels (Table 3). Moreover, internal migrants were less likely to have doubts about vaccination than local residents, which may be because internal migrants in China have lower education and income level [46]. However, their lower awareness of health problems and less health education [47,48,49] might lead to the less probability to have professional sources than local residents (Table 1). Therefore, health communication interventions should especially target more educated and wealthier caregivers and internal migrants to increase their exposure to information from professional sources, and should use the media to disseminate more evidence-based vaccination information.

Our study also showed that caregivers’ demographic characteristics were associated with vaccine confidence/hesitancy. First, females had lower confidence in vaccine safety than males. Generally, mothers care more about children’s health issues than fathers [50], so they may be more likely to have concern about the vaccination when facing vaccine incidents. A study in China suggested that females’ confidence towards vaccination decreased more than males’ after the Changchun Changsheng vaccine incident [29]. Second, caregivers aged 30–35 years were more likely to be hesitant to vaccination than those under 30 years old, while those aged >40 years were less likely to be hesitant. We found caregivers >40 years old had less information sources and were less prone to have media or interpersonal sources but more research is needed to explore the reasons why caregivers aged 30–35 years were more hesitant to vaccination. Last, caregivers who had Buddhism or other religions had a higher prevalence of vaccine hesitancy than those with no religious beliefs. Buddhism or other religions in China may be incompatible with vaccination. Previous research showed that 25% of people report religious incompatibility to vaccines in the Southeast Asian and Western Pacific regions where Buddhism is widespread [4]. Further research is needed to investigate the compatibility between religious beliefs and vaccination among Chinese people and explore its reasons.

Interestingly, our study showed a high prevalence of vaccine hesitancy (60%) as well as high rates of confidence in vaccine safety (82.7%), vaccine effectiveness (88.3%), and healthcare professionals (92.1%). Vaccine confidence issues are not the only factors that influence people’s vaccine hesitancy. According to the World Health Organization, vaccine hesitancy is determined by broad and complex factors, such as vaccine confidence, convenience, and complacency [51]. Broadly, there are three domains of vaccine hesitancy determinants: contextual influences, individual, and group influences, and vaccine and vaccination-specific issues [2]. In this model, communication and media environment is a key issue of contextual influences [2]. Our results also illustrated that media and interpersonal information sources are associated with higher prevalence of vaccine hesitancy, whereas the two information sources have no influence on vaccine confidence. Therefore, high rates of vaccine confidence can co-exist with high level of vaccine hesitancy.

Our study had certain limitations. First, there may be a degree of recall bias as questionnaires were self-reported and collected information on past childhood vaccination behavior. Participants may misunderstand some questions in the self-administered questionnaires, for example, what the accurate definition of primary information sources was, and we attempted to address this problem with assistance from interviewers. Second, our sampling methodology may lead to a selection bias, as half the participants were recruited via vaccination clinics. Caregivers who take children to vaccination clinics may have higher vaccine confidence and lower vaccine hesitancy than those who do not present at vaccination clinics. We also could not identify the content of information caregivers accessed through the different information sources. Besides, we did not explore the dose response within each type of information sources, such as three sources (health professionals, health education activities, and professional books) within professional information source, although we assessed the influence of numbers of information sources. Finally, causal inference from these findings cannot be drawn due to the cross-sectional nature of the study.

## 5. Conclusions

Caregivers who reported professional information sources had higher confidence and lower hesitancy towards vaccines, whereas caregivers who reported multiple information sources or primarily receiving vaccination information from media sources had higher vaccine hesitancy. Only 69% of caregivers reported health professionals as their primary information sources. To address vaccine hesitancy, it is essential to promote universal access to professional information sources by motivating primary care professionals to be involved in health communication on vaccination, and to make effective use of the media to disseminate evidence-based information and clarify vaccination misinformation. Health communication interventions could target internal migrants, and more educated and wealthier caregivers.

## Figures and Tables

**Table 1:** Caregiver characteristics, number, and types of vaccine-related information sources in China, N = 2122.

Variables	Total, N (column%)	Number of Information Sources		Type of Information Sources					
				Professional Sources		Media Sources		Interpersonal Sources	
		Mean	*p*-Value	N (row%)	*p*-Value	N (row%)	*p*-Value	N (row%)	*p*-Value
Total	2122 (100%)	2.3	-	1728 (81.4)	-	1346 (63.4)	-	546 (25.7)	-
Region									
Shaanxi province	857 (40.4)	2.2	0.047	678 (79.1)	0.071	551 (64.3)	0.243	224 (26.1)	0.620
Anhui province	855 (40.3)	2.3		707 (82.7)		525 (61.4)		211 (24.7)	
Shenzhen city	410 (19.3)	2.4		343 (83.7)		270 (65.9)		111 (27.1)	
Living residence									
Urban	1294 (61.0)	2.3	<0.001	1068 (82.5)	0.103	853 (65.9)	0.003	337 (26.0)	0.680
Rural	828 (39.0)	2.2		660 (79.7)		493 (59.5)		209 (25.2)	
Gender									
Male	496 (23.4)	2.2	0.009	387 (78.0)	0.026	312 (62.9)	0.781	115 (23.2)	0.139
Female	1626 (76.6)	2.3		1341 (82.5)		1034 (63.6)		431 (26.5)	
Age group (years)									
<30	766 (36.1)	2.3	0.002	618 (80.7)	0.906	501 (65.4)	0.026	184 (24.0)	0.333
30–35	853 (40.2)	2.3		696 (81.6)		537 (63.0)		228 (26.7)	
35–40	266 (12.5)	2.4		219 (82.3)		177 (66.5)		77 (29.0)	
>40	237 (11.2)	2.1		195 (82.3)		131 (55.3)		57 (24.1)	
Religious beliefs									
None	1956 (92.2)	2.3	0.399	1597 (81.7)	0.385	1244 (63.6)	0.580	500 (25.6)	0.543
Buddhism or others	166 (7.8)	2.2		131 (78.9)		102 (61.5)		46 (27.7)	
Education level									
Middle school or below	391 (18.4)	1.9	<0.001	325 (83.1)	0.511	184 (47.1)	<0.001	88 (22.5)	0.002
High school	411 (19.4)	2.2		329 (80.1)		251 (61.1)		96 (23.4)	
Three-year technical college	575 (27.1)	2.4		475 (82.6)		385 (67.0)		133 (23.1)	
Bachelor degree or above	745 (35.1)	2.4		599 (80.4)		526 (70.6)		229 (30.7)	
Annual household income (1000 RMB)									
<20	296 (14.0)	2.1	<0.001	237 (80.1)	0.024	187 (63.2)	<0.001	59 (19.9)	<0.001
20–50	425 (20.1)	2.2		370 (87.1)		233 (54.8)		76 (17.9)	
50–100	619 (29.2)	2.3		497 (80.3)		382 (61.7)		180 (29.1)	
100–200	479 (22.6)	2.3		382 (79.8)		323 (67.4)		132 (27.6)	
>200	301 (14.2)	2.4		240 (79.7)		219 (72.8)		99 (32.9)	
Registered residence									
Local residents	1728 (81.4)	2.3	0.896	1421 (82.2)	0.047	1091 (63.1)	0.556	431 (24.9)	0.082
Internal migrants	394 (18.6)	2.3		307 (77.9)		255 (64.7)		115 (29.2)	

**Table 2:** Comparison of vaccine confidence and hesitancy by caregivers’ reported information sources in China, N = 2122.

Variables		Confident in Vaccine Safety, N (row%)		Confident in Vaccine Effectiveness, N (row%)		Confident in Health Professionals, N (row%)		Hesitant to Vaccination, N (row%)	
		Agree	Disagree	Agree	Disagree	Agree	Disagree	Yes	No
Total		1754 (82.7)	368 (17.3)	1870 (88.3)	249 (11.8)	1953 (92.1)	168 (7.9)	1273 (60.0)	849 (40.0)
Number of primary information sources	Single source	497 (83.8)	96 (16.2)	526 (88.9)	66 (11.2)	551 (92.9)	42 (7.1)	272 (45.9)	321 (54.1)
	Two sources	312 (82.1)	68 (17.9)	341 (89.7)	39 (10.3)	353 (92.9)	27 (7.1)	236 (62.1)	144 (37.9)
	Three or more sources	945 (82.3)	204 (17.8)	1003 (87.5)	144 (12.6)	1049 (91.4)	99 (8.6)	765 (66.6)	384 (33.4)
	*p*-value	0.681		0.421		0.428		<0.001	
Professional sources	Yes	1458 (84.4)	270 (15.6)	1537 (89.1)	188 (10.9)	1603 (92.8)	124 (7.2)	1007 (58.3)	721 (41.7)
	No	296 (75.1)	98 (24.9)	333 (84.5)	61 (15.5)	350 (88.8)	44 (11.2)	266 (67.5)	128 (32.5)
	*p*-value	<0.001		0.011		0.008		0.001	
Media sources	Yes	1088 (80.8)	258 (19.2)	1170 (87.1)	174 (13.0)	1224 (91.0)	121 (9.0)	899 (66.8)	447 (33.2)
	No	666 (85.8)	110 (14.2)	700 (90.3)	75 (9.7)	729 (93.9)	47 (6.1)	374 (48.2)	402 (51.8)
	*p*-value	0.003		0.024		0.016		<0.001	
Interpersonal sources	Yes	433 (79.3)	113 (20.7)	468 (85.7)	78 (14.3)	489 (89.6)	57 (10.4)	371 (68.0)	175 (32.1)
	No	1321 (83.8)	255 (16.2)	1402 (89.1)	171 (10.9)	1464 (93.0)	111 (7.1)	902 (57.2)	674 (42.8)
	*p*-value	0.016		0.033		0.011		<0.001	

**Table 3:** Determinants of vaccine confidence and hesitancy using logistic regression.

Variables (Reference Group)	Confident in Vaccine Safety	Confident in Vaccine Effectiveness	Confident in Health Professionals	Hesitant to Vaccination
Number of information sources (Single source)				
Two sources	0.94 (0.69–1.29)	1.21 (0.83–1.76)	1.20 (0.75–1.90)	1.73 ** (1.22–2.47)
Three or more sources	0.94 (0.64–1.37)	1.03 (0.64–1.65)	1.15 (0.69–1.91)	1.91 ** (1.36–2.68)
Professional sources	1.68 * (1.09–2.59)	1.30 (0.80–2.12)	1.32 (0.79–2.23)	0.63 ** (0.45–0.87)
Media sources	0.84 (0.60–1.17)	0.78 (0.54–1.10)	0.72 (0.46–1.14)	1.47 ** (1.19–1.83)
Interpersonal sources	0.88 (0.66–1.18)	0.77 (0.55–1.08)	0.68 (0.46–1.01)	1.21 (0.94–1.56)
Region (Shenzhen city)				
Shaanxi province	1.51 ** (1.14–1.99)	1.01 (0.71–1.43)	1.01 (0.60–1.70)	1.18 (0.91–1.53)
Anhui province	1.42 ** (1.09–1.83)	1.03 (0.77–1.39)	1.34 (0.78–2.30)	1.15 (0.86–1.53)
Rural residence (Urban)	0.91 (0.73–1.14)	1.01 (0.80–1.28)	0.99 (0.74–1.32)	0.86 (0.71–1.05)
Female (Male)	0.66 * (0.48–0.91)	0.85 (0.64–1.12)	0.74 (0.51–1.06)	1.11 (0.83–1.48)
Age group (<30, years)				
30–35	0.84 (0.65–1.07)	0.98 (0.73–1.31)	1.06 (0.74–1.53)	1.52 ** (1.22–1.90)
35–40	0.77 (0.51–1.14)	1.26 (0.95–1.68)	0.97 (0.59–1.61)	0.99 (0.74–1.33)
>40	0.93 (0.61–1.42)	0.82 (0.50–1.33)	1.88 (0.85–4.17)	0.54 ** (0.38–0.76)
Having Buddhism or other religions (None)	0.66 (0.42–1.04)	0.66 (0.40–1.09)	0.67 (0.38–1.18)	1.70 ** (1.16–2.49)
Education level (Middle school or below)				
High school	0.89 (0.62–1.29)	0.98 (0.64–1.48)	1.20 (0.63–2.26)	0.97 (0.73–1.30)
Three-year technical college	0.92 (0.63–1.33)	0.77 (0.51–1.15)	0.72 (0.45–1.14)	1.33 * (1.04–1.72)
Bachelor degree or above	1.11 (0.73–1.67)	0.98 (0.62–1.54)	0.68 (0.37–1.28)	1.14 (0.88–1.48)
Annual household income (<20, 1000 RMB)				
20–50	1.24 (0.87–1.76)	1.44 (0.95–2.19)	1.13 (0.60–2.13)	0.98 (0.70–1.36)
50–100	1.00 (0.72–1.40)	1.28 (0.87–1.89)	1.29 (0.75–2.20)	1.10 (0.78–1.55)
100–200	0.76 (0.54–1.06)	1.34 (0.90–1.97)	1.09 (0.62–1.92)	0.88 (0.63–1.24)
>200	0.65 * (0.44–0.94)	0.93 (0.61–1.43)	0.84 (0.42–1.67)	1.22 (0.83–1.78)
Internal migrants (Local residents)	0.77 (0.58–1.03)	1.01 (0.72–1.42)	1.19 (0.69–2.04)	0.78 * (0.63–0.98)
Observations	2120	2117	2119	2120

Adjusted odds ratios and 95% confidence intervals were reported. ** *p* < 0.01, * *p* < 0.05.

## Data Availability

The data presented in this study are available on request from the corresponding author. The data are not publicly available due to program agreement.
